# Case Report: *RAB10-ALK*: A Novel *ALK* Fusion in a Patient With Gastric Cancer

**DOI:** 10.3389/fonc.2021.645370

**Published:** 2021-02-22

**Authors:** Zhengqi Wen, Dun Xiong, Shurong Zhang, Jiankun Liu, Bitao Li, Raomei Li, Hushan Zhang

**Affiliations:** ^1^ Department of Oncology, The First Affiliated Hospital of Kunming Medical University, Kunming, China; ^2^ Department of Oncology, The People’s Hospital of Puer City, Puer, China; ^3^ Department of Gastroenterology, The 920th Hospital of the Joint Logistics Support Force, PLA, Kunming, China; ^4^ Department of Prevention and Health Care, The First Affiliated Hospital of Kunming Medical University, Kunming, China; ^5^ The Medical Department, 3D Medicines Inc., Shanghai, China

**Keywords:** anaplastic lymphocyte kinase fusion, anaplastic lymphocyte kinase-tyrosine kinase inhibitor, gastric cancer, next generation sequencing, cancer

## Abstract

Gastric cancer is one of the most common cancers, while the current treatment options for gastric cancer are relatively scarce due to insufficient understanding of molecular characteristics and subtypes of gastric cancer. Different gene rearrangements of anaplastic lymphocyte kinase (*ALK*) have been reported in several types of cancer, especially in NSCLC. The first-generation *ALK* tyrosine kinase inhibitor (TKI) crizotinib, second-generation (ceritinib, alectinib, and brigatinib) and third-generation (lorlatinib) *ALK*-TKIs have been widely used for NSCLC patients with *ALK* rearrangement. However, little was reported about *ALK* mutation in gastric cancer (GC). Here we identified a novel form of *ALK* fusion, a case of GC with *RAB10-ALK* fusion, and this is the first report of *ALK* fusion in gastric cancer.

## Introduction

In recent years, *ALK* tyrosine kinase inhibitor (TKI) therapy has received great attention in solid tumors such as non-small cell lung cancer. However, few was reported about gene rearrangement of *ALK* in GC. Here we firstly present a case of GC with *RAB10-ALK* fusion, which is the first report of *ALK* fusion in GC.

## Case Presentation

A 66-year-old male patient who is a minority (Lisu nationality) in Yunnan, China, was admitted to the hospital due to abdominal pain. The patient has no history of smoking and drinks alcohol occasionally. He has a history of hemorrhoids for many years without special treatment, has no history of infectious diseases such as hepatitis, tuberculosis, malaria, has no history of hypertension, and has no history of surgery. Both his parents are dead; the cause of death is unknown. He has no family history of infectious diseases and genetic diseases. The patient has symptoms of dry mouth, bitter mouth, no nausea, no acid reflux, no melena, mucus pus and blood in the stool, no fever. The mental state is fair, the physical strength is decreased, the appetite is poor, and the sleep is poor. There was no swelling and tenderness of superficial lymph nodes throughout the body.

**Figure 1:**
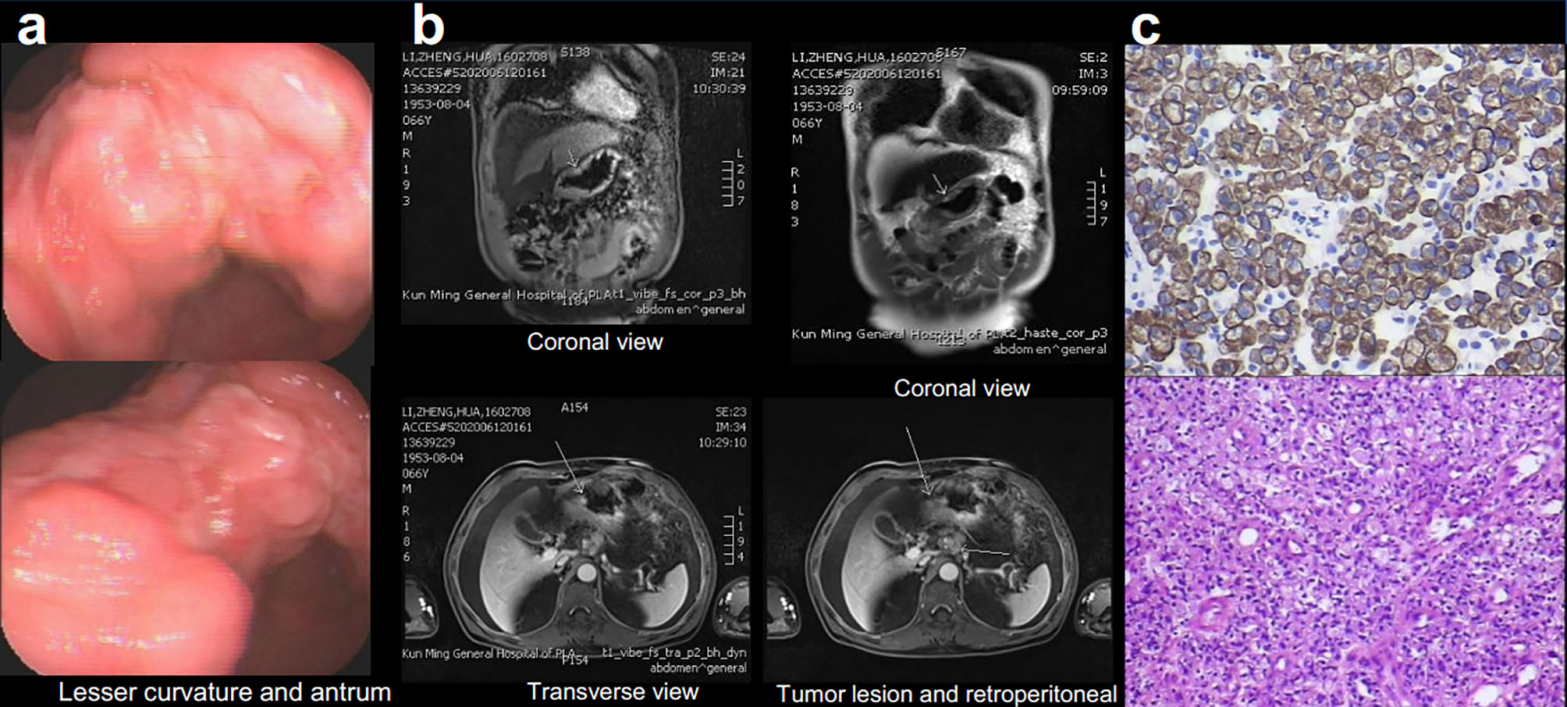
Gastroscopy **(A)**, Magnetic Resonance Imaging (MRI) **(B)** displayed the tumor lesion, pathological diagnosis **(C)**, Immunohistochemical (IHC) staining for cytokeratin (CK) 6 and hematoxylin–eosin staining (HE).

**Figure 2:**
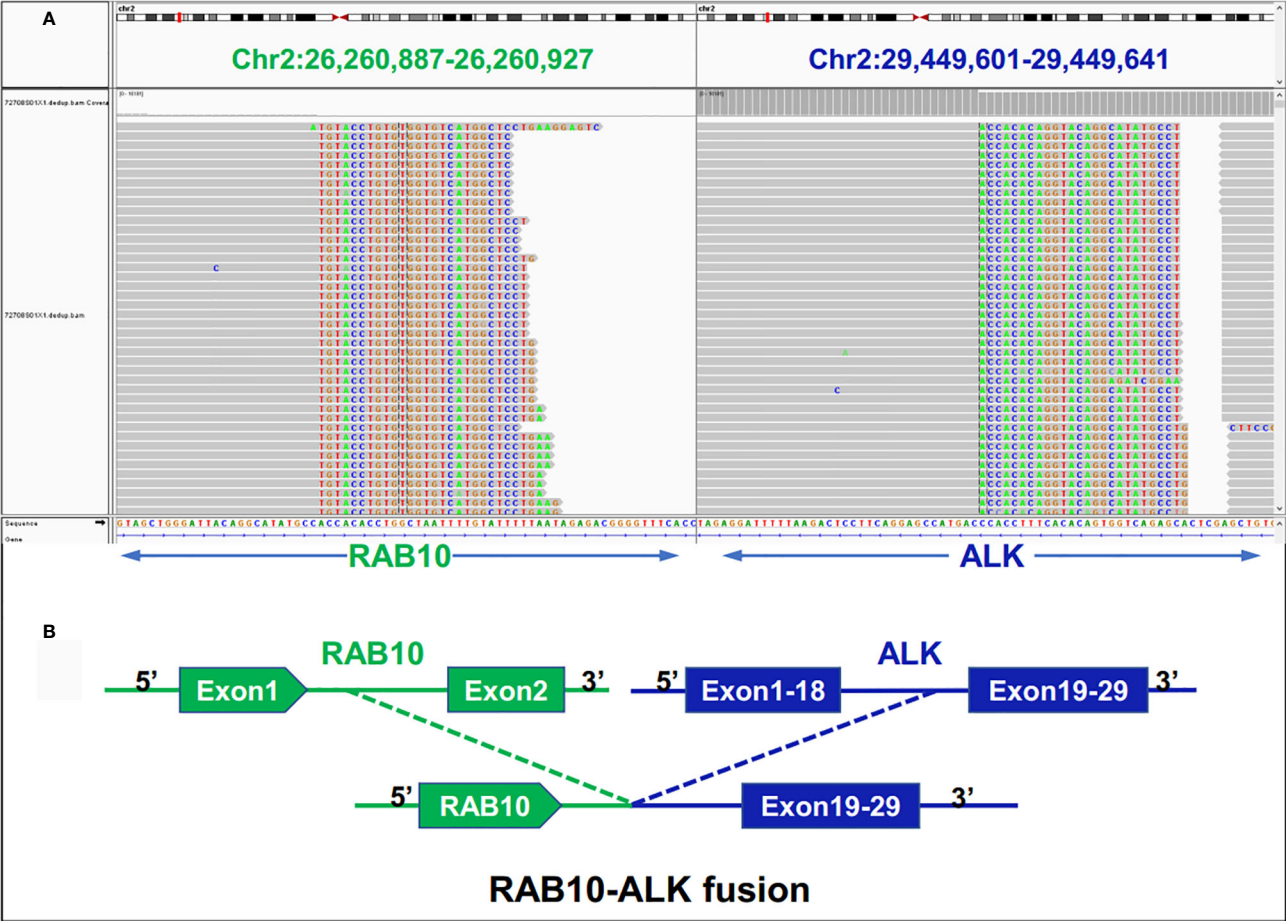
Next-generation sequencing findings for the tumor tissue samples of the patient with gastric cancer. **(A)** *RAB10-ALK* fusion was validated manually using the Integrated Genomics Viewer (IGV); **(B)** a novel intergenic region between RAB10 and *ALK* exon 20 fusion variant was identified.

**Figure 3:**
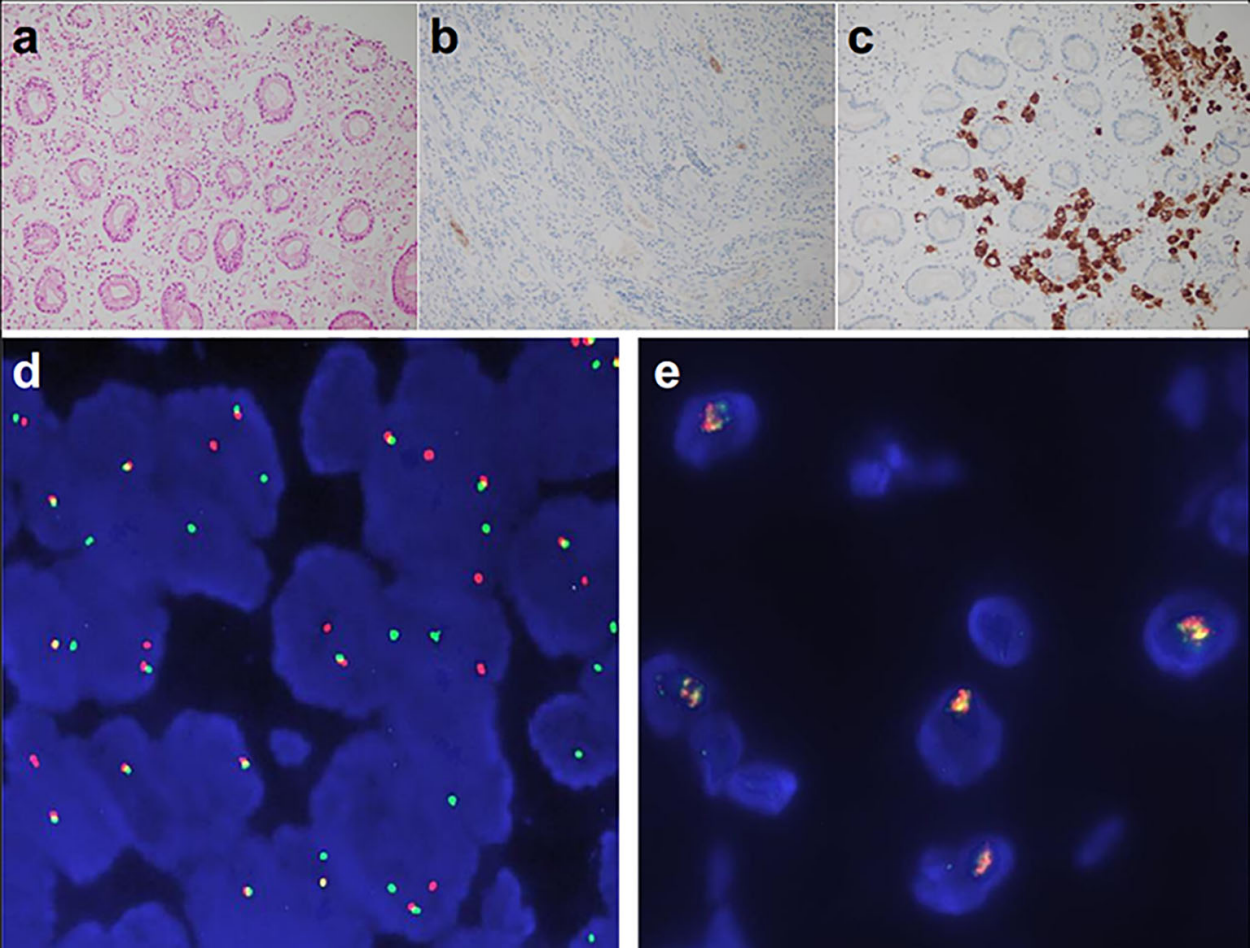
*ALK* detected by Ventana-D5F3 IHC assay and FISH. **(A)** HE staining (200×); **(B)** *ALK* negative control 200×; **(C)** *ALK* IHC staining of the patient (200×), **(D)** EML4-*ALK* fusion positive control; **(E)** *ALK* detection of the patients, amplification of ALK was displayed (16%).

Examination after admission showed a significant increase in serum biomarkers, including carcinoembryonic antigen, carbohydrate antigen, carbohydrate antigen CA199, carbohydrate antigen CA125, Cytokeratin-19-fragment CYFRA21-1. Gastroscopy revealed unevenness, ulcers, congestion, and edema at the lesser curvature and antrum of stomach (**Figure 1A**). Magnetic resonance examination displayed a large amount of fluid in the abdominal cavity and multiple lymph nodes adjacent to the abdominal aorta and enlarged (**Figure 1B**). Pathological examination revealed poorly differentiated adenocarcinoma of the stomach (signet ring cell carcinoma) (**Figure 1C**). In addition, the needle biopsy specimen was subjected to next generation sequencing (NGS) analysis in a laboratory accredited by the College of American Pathologists (CAP) and Clinical Laboratory Improvement Amendment (CLIA) (3D Medicines Inc., Shanghai, China), covering whole exon regions of 733 cancer related genes; as a result, fusion of *RAB10-ALK* with frequency of 15.23% and *ALK* amplification with frequency of 16% were detected in the stomach tissue of the patient (**Figures 2A, B**). In addition, several other genetic variants were also found, including KDM6A, TP53 mutation, and increased copy number of gene FGF19, BTK, IRS2, FGF3, EMSY(C11orf30), FGF4. Furthermore, immunocytochemistry (IHC) and fluorescence *in situ* hybridization (FISH) were also performed to verify the above mutation (**Figures 3A–E**). Immunohistochemical staining (Ventana Medical Systems, Tucson, AZ) showed an increased signal of *ALK* expression (**Figure 3C**), and *ALK* amplification was also verified by FISH (**Figure 3E**). However, the results of FISH excluded the common *ALK* fusion form EML4-*ALK* fusion (**Figures 3D, E**).

## Discussion

*ALK* is one of the membrane-bound receptor tyrosine kinases, which consist of an extra-cellular ligand binding domain, a single transmembrane domain, and a cytoplasmic tyrosine kinase domain.

In *ALK* fusions such as EML4-*ALK*, the amino-terminal fusion partner is fused to the intracellular tyrosine kinase domain of *ALK*, resulting in activation of downstream signaling. *ALK* signals activate numerous downstream pathways, including PI3K–Akt activation, MEKK2/3/MEK5/ERK5 pathway, RAS-MAPK, CRKL-C3G-RAP1, JAK-STAT and JUN pathway (1). In fact, almost all the *ALK* fusion proteins usually retain the cytoplasmic tyrosine kinase domain of *ALK* at the C-terminus, while the N-terminus is composed of a different protein. Several clinical trials have now confirmed that patients with *ALK* positive NSCLC can benefit from treatment with *ALK*-TKIs such as crizotinib, ceritinib, alectinib, brigatinib, and lorlatinib (2). Recent retrospective clinical researches in *ALK* positive NSCLC suggest different clinical outcomes based on the specific *ALK* fusion protein. That is, different *ALK* fusion form can mediate different downstream signaling and may exhibit different sensitivity to *ALK* tyrosine kinase inhibitors (TKIs) (2). The NGS method has expanded the different fusion partners identified in *ALK* positive NSCLC to 90, including Potential Fusion Partners RAB10 (3). However, except for EML4-*ALK*, which is the most prevalent *ALK* gene rearrangement in the *ALK*-positive NSCLC patients, there are few in-depth studies on most of other *ALK* fusions. The currently known evidence about *ALK* fusion proteins mainly originates from some of clinical cohorts of patients with *ALK*-positive NSCLC. Here, we firstly report the *RAB10-ALK* fusion and *ALK* amplification that are identified in patients with gastric cancer. This is the first report of an *ALK* fusion case in gastric cancer, and this is a novel type of *ALK* fusion. Regrettably, the patient refused to use the drug *ALK*-TKI of cross-indications, such as crizotinib, ceritinib *etc*.

Different *ALK* inhibitors have been used to the majority of *ALK*-positive patients, and all have shown a certain effect in controlling disease progression, especially in NSCLC (4). Well use of different *ALK*-TKIs will benefit the patient more. As reported, according to the different *ALK* mutation sites detected by NGS, a female patient with metastatic *ALK* rearrangement NSCLC received treatment of different *ALK*-TKIs, and finally the patient survived more than 48 months (5). However, rare cases of *ALK* mutations have been observed among GC patients, and there is no applications of *ALK*-TKIs in the GC treatment at present. For the efficacy of *ALK*-TKIs in NSCLC, crizotinib was also recommended to the patient reported in this case, but he refused because of his own reasons.

GC is one of the most common cancers worldwide. Although the incidence of GC has been steadily declining in the past few decades, due to the current lack of understanding of GC molecular characteristics and subtypes, the current treatment options for gastric cancer patients are relatively monotonous. Previous studies on the gene fusion mutations in gastric or signet ring adenocarcinoma are few, which may be related to the usual use of methods other than NGS in the past. For example, in 42 cases of signet ring cell carcinoma of the gastrointestinal tract, two different *ALK* antibody based IHC did not detect the “possibly positive” cases with *ALK* translocation detected by FISH (6). With the application of NGS technology and other genomic technologies, GC is currently being studied and typed in more detail at the molecular level. Gene fusions were also found in gastric cancer through NGS technology. Some researchers conducted whole-genome sequencing on 32 pairs of gastric signet ring cell carcinoma samples and found frequent CLDN18-ARHGAP26/6 fusion protein (25%), which was associated with a poor prognosis (7). Accordingly, based on the wider application of NGS detection technology, GC can be diagnosed and treated more accurately in the future. *ALK* rearrangements have been reported in several types of cancer, such as NSCLC, breast cancer, renal cell carcinoma (RCC), diffuse large B-cell lymphoma (DLBCL), serous ovarian carcinoma (SOC), inflammatory myofibroblastic tumor (IMT), renal medulla carcinoma (RMC), colon cancer, and to a lesser extent, esophageal squamous cell carcinoma (ESCC) (8); however, data on *ALK* rearrangement in GC is rare now.

About 90 different *ALK* fusion partners have been already identified, such as EML4, TPM-3/-4, TFG, CLTC, PRKAR1A, LMNA, KIF5B, RANBP2, FN1 (3). Although these partner genes have been described for *ALK*, little data is currently addressed on how different fusion partners affect the efficacy of *ALK*-TKIs. Therefore, the therapy of *ALK* positive cancers is currently determined regardless of which fusion partner is present. However, with the development of NGS technology and the advancement of precision medicine, more fusion partners will be identified and clinical evidence will be accumulated; based on these, different clinical strategies will be applied to patients with different *ALK* fusions. RAB10 is a novel *ALK* fusion partner that has been associated with cancer. RAB10 is a key regulator of endocytic recycling, belongs to the RAS oncogene superfamily, and it is reported to be an oncogene in HCC and is associated with poor prognosis (9). *ALK* fusion usually leads to abnormal activation of the *ALK* kinase domain and induces the activation of downstream signal transduction, leading to the growth of tumor cells. Since *RAB10-ALK* is a novel fusion mode, whether the domains reserved in RAB10 may contribute to the activation of *RAB10-ALK* remains unknown. Therefore, it is necessary to further research and verify the function of *RAB10-ALK* and the response to TKI.

## Conclusion

This case provides a new reference for understanding *ALK* fusion mutations, discovers new molecular characteristics of GC patients, and provides the possibility for the future application of *ALK*-TKIs in GC patients. NGS can be performed as a routine test to explore more opportunities for treating GC patients.

## Data Availability Statement

The original contributions presented in the study are included in the article/supplementary material, further inquiries can be directed to the corresponding author/s.

## Ethics Statement

The studies involving human participants were reviewed and approved by the Ethics Committee of the First Affiliated Hospital of Kunming Medical University. The patients/participants provided their written informed consent to participate in this study. Written informed consent was obtained from the individual(s) for the publication of any potentially identifiable images or data included in this article.

## Author Contributions

HZ and ZW put forward the content of the paper. HZ wrote the manuscript. DX, BL, JL, RL and SZ reviewed literature and clinical data. All authors contributed to the article and approved the submitted version.

## Conflict of Interest

HZ is employed by the company 3D Medicines Inc.

The remaining authors declare that the research was conducted in the absence of any commercial or financial relationships that could be construed as a potential conflict of interest.

## Abbreviations

*ALK*, anaplastic lymphocyte kinase; CAP, College of American Pathologists; CLIA, Clinical Laboratory Improvement Amendment; FISH, fluorescence *in situ* hybridization; GC, gastric cancer; IHC, immunocytochemistry; NGS, next-generation sequencing; NSCLC, non-small cell lung cancer; TKI, tyrosine kinase inhibitor.
